# Gait Improvement After Distal Femoral Osteotomy for Permanent Patellar Dislocation Following Femoral Lengthening in an Adolescent With Achondroplasia: A Case Report

**DOI:** 10.7759/cureus.82172

**Published:** 2025-04-13

**Authors:** Shusuke Nojiri, Shinya Ishizuka, Yuki Fugane, Riku Arai, Chiaki Terai, Kenichi Mishima

**Affiliations:** 1 Department of Rehabilitation, Nagoya University Hospital, Nagoya, JPN; 2 Department of Orthopaedic Surgery, Nagoya University Graduate School of Medicine, Nagoya, JPN

**Keywords:** around knee osteotomy, gait training, genu valgum, limb lengthening, rehabilitation, skeletal dysplasia, valgus knee deformity

## Abstract

In patients with skeletal dysplasia, certain disease-specific and treatment-related characteristics may affect functional outcomes following orthopedic surgery. We report a case of valgus deformity with permanent patella dislocation after femoral lengthening treated with distal femoral osteotomy (DFO), in which improvement in gait ability was achieved during the postoperative rehabilitation course. A 15-year-old Japanese boy with achondroplasia (ACH) had undergone tibial and femoral lengthening. During the femoral lengthening, valgus deformity and the accompanying permanent patella dislocation on both sides appeared; therefore, a staged bilateral knee osteotomy was scheduled. The preoperative status included a decreased walking speed of 0.68 m/s with a requirement for crutches. The surgery was performed bilaterally with a six-week interval and included DFO, lateral retinacular release, and medial patellofemoral ligament (MPFL) reconstruction. After a six-week non-weight-bearing period in each limb, the patient was able to walk independently with crutches; however, lateral asymmetry and lack of bimodal pattern, in addition to a decreased speed of 0.60 m/s, were noted by gait analysis. Additional rehabilitation programs, including gait training using real-time feedback of absolute load, were implemented to increase lower-limb loads and normalize the gait cycle. After a further nine weeks of inpatient rehabilitation, improvements in lateral asymmetry and load pattern were observed with a speed of >1.0 m/s without a walking aid. These results highlight that perioperative rehabilitation combined with DFO can effectively improve physical function in ACH patients with severe functional impairments and characteristic limb deformities.

## Introduction

Achondroplasia (ACH) is the most common skeletal dysplasia characterized by short-limbed short stature with rhizomelia, due to a mutation in the FGFR3 gene [1]. Bone lengthening using an external fixator has been indicated for children and adolescents with ACH to avoid short stature-related decreases in physical function and quality of life in adulthood [2,3]. Although bone lengthening has the advantage of alleviating functional limitations associated with short limbs, it involves minor and major complications such as pin-tract infections, joint stiffness/instability, neurologic injury, refractures, and lower limb malalignment [4-6]. In particular, patients with inherent joint laxity are at increased risk of instability in adjacent joints during lengthening due to imbalances in tension between muscles, tendons, and growing bones. Subluxation and dislocation of the knee or hip have been observed in patients with congenital femoral deficiency who underwent femoral lengthening [7]. Lateral patellar instability may result from various patient-specific factors, such as marked osseous incongruity, ligamentous laxity, and abnormal limb alignment [8]. Permanent patellar dislocation has been observed in some ACH patients with significant trochlear dysplasia, whereas acute patellar dislocation during bone lengthening has rarely been documented in the literature [9].

Management of lateral patellar instability should be based on a thorough assessment of anatomical risk factors and individualized treatment planning. Medial patellofemoral ligament (MPFL) reconstruction with anatomical placement of the femoral tunnel has become a reliable surgical option in most cases of patellar instability [10,11]. In cases exhibiting lateral retinacular tightness, lateral retinacular release or lengthening should be considered and combined with other stabilizing procedures [12,13]. When valgus knee malalignment is a contributing factor, distal femoral osteotomy (DFO) is performed to correct alignment and reduce lateral stress, as the increased Q angle exerts a greater lateralizing force on the patella [14].

In this paper, we report a case of an adolescent with ACH who underwent MPFL reconstruction combined with DFO for irreducible bilateral patellar dislocation occurring during femoral lengthening. Regardless of poor preoperative physical function and disease-specific limb deformities, improvements in range of motion (ROM) and gait ability were achieved through postoperative rehabilitation.

## Case presentation

Patient information

**Figure 1:**
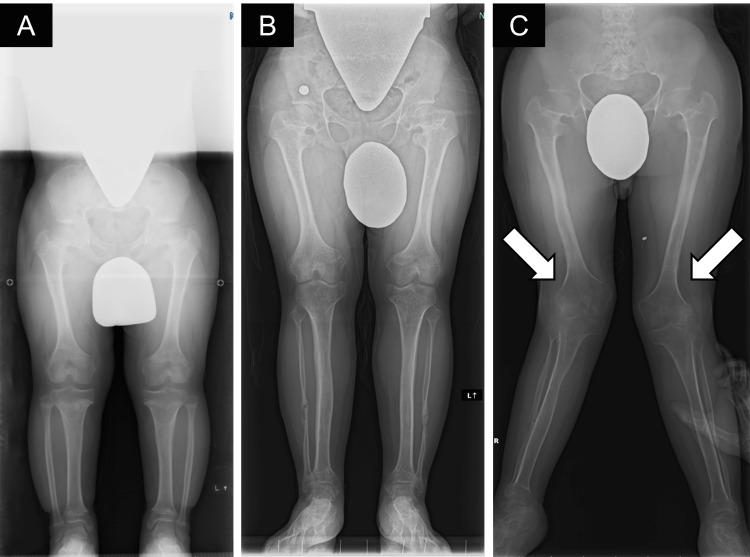
Preoperative X-rays A) Before tibia lengthening at the age of 10; B) Before femoral lengthening at the age of 13; C) After femoral lengthening. After femoral lengthening, valgus knee deformity was observed.

A Japanese boy with ACH underwent tibial lengthening with the use of a unilateral external fixator (DynaFix rail system; EBI LP, Parsippany, New Jersey, USA) between the ages of 10 and 11 years (Figure 1A; Figure 1B). Uninterrupted lengthening of the tibia was performed for four months at a rate of up to 1 mm per day, resulting in a 10 cm increase in length. Eleven months after the procedure, the fixators were removed based on the radiographic appearance of the regenerated bones. No signs and/or symptoms suggestive of patellofemoral instability or maltracking were observed at that time or regular visits thereafter. He had restored acceptable knee flexion of 130 degrees with full extension by the time of femoral lengthening.

**Figure 2:**
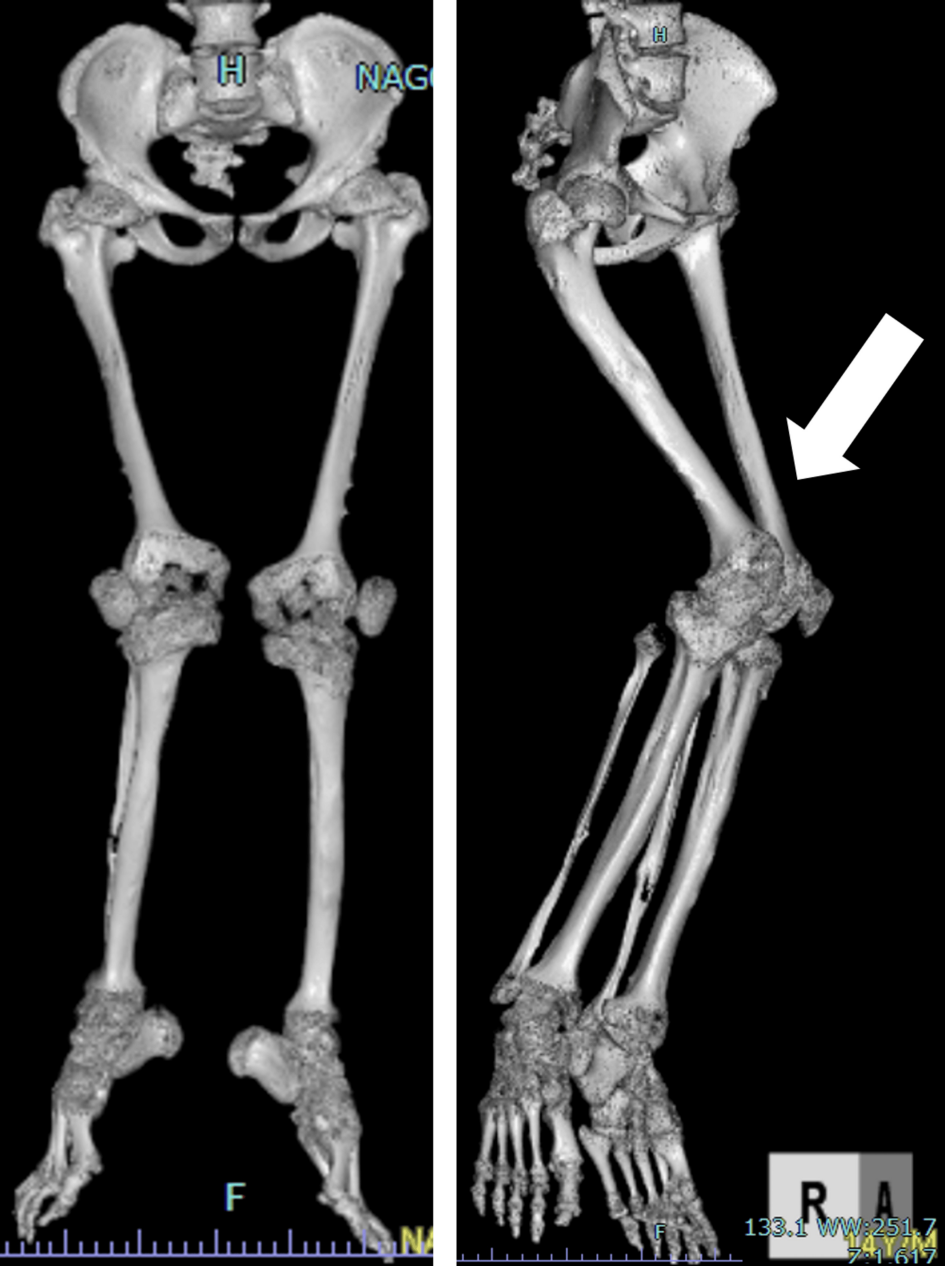
Preoperative CT images As pointed to by the white arrow, knee flexion contracture was observed in addition to valgus knee deformity and permanent patellar dislocation.

The femoral lengthening was performed in the same manner as the tibial lengthening at 13 years of age. The femur was continuously distracted for two months until the occurrence of left knee pain, followed by intermittent halting of lengthening due to worsening knee flexion-valgus-external rotational contractures. Once he could not walk with crutches, three months after the beginning of lengthening, distraction ceased. The total amount of femoral lengthening achieved was 74 and 69 mm for the right and left sides, respectively. A high index of suspicion for patellar dislocation prompted us to obtain knee radiographs, revealing complete dislocation of the left patella and a lateralized right patella perched on the lateral femoral condyle. One month later, we performed Z-lengthening of the iliotibial band and fractional lengthening of the distal rectus femoris tendon with the fixator and half pins left in situ; however, lateral displacement of the patella became unchanged. Weight-bearing and ROM exercises as tolerated resumed during dynamization. At the time of pin removal, one year after the initiation of distraction, we performed detachment of the vastus lateralis insertion from the patella, selective release of the lateral structures of the patellofemoral joint, and fractional lengthening of the medial hamstrings; however, these interventions had little effect on the patellar malposition (Figure 1C; Figure 2). After pin removal, he started wearing a custom-made long leg brace at night to gradually correct residual knee flexion contractures.

**Figure 3:**
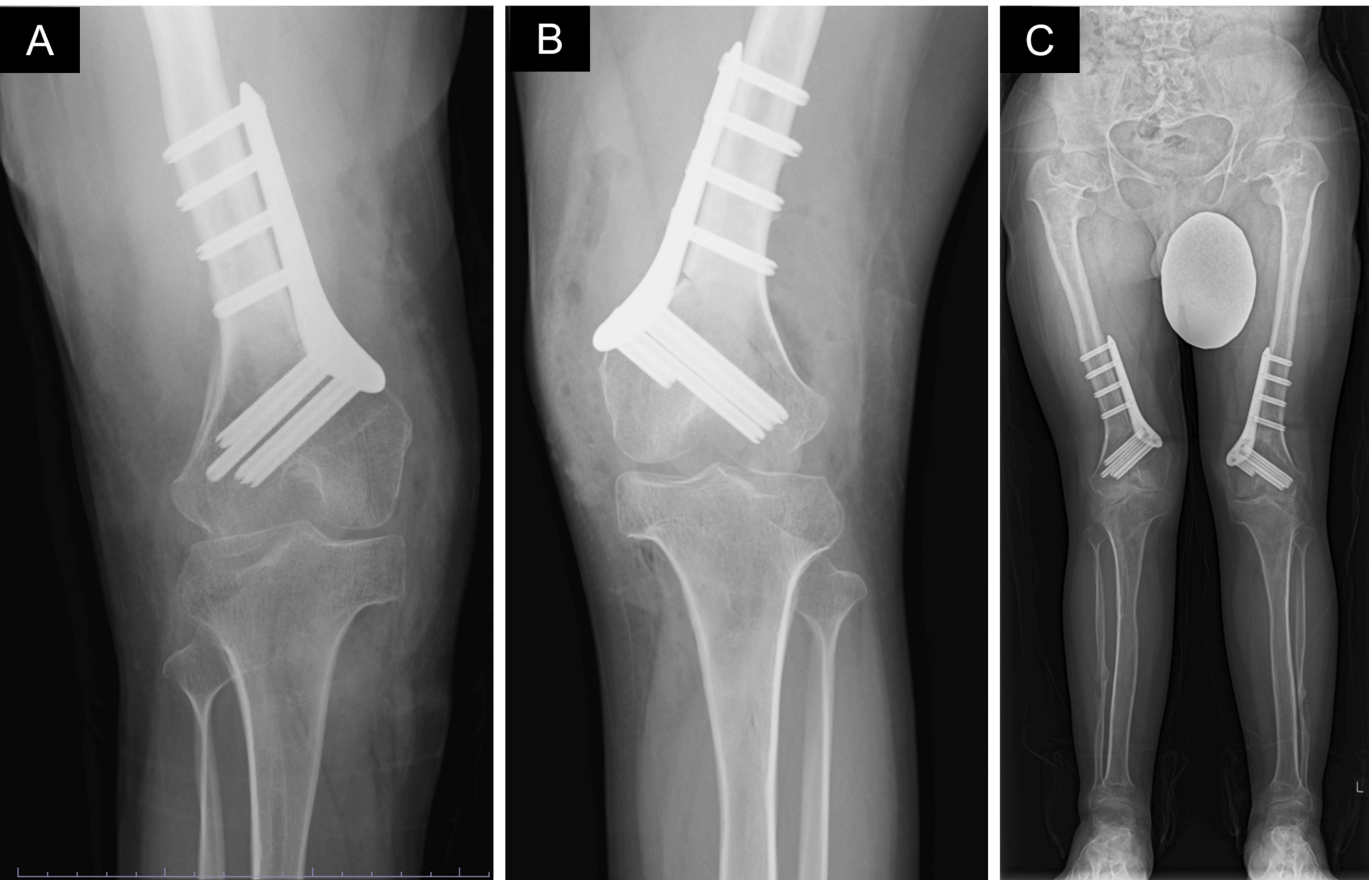
Postoperative X-ray images A) Right knee immediately after the operation; B) Left knee immediately after the operation; C) Standing posture four months after right knee surgery.

Once he regained independent walking ability with crutches, a staged bilateral DFO combined with MPFL reconstruction was scheduled. An extensive lateral patellar retinacular release was performed to achieve stable reduction and normal tracking of the left patella without the need for manual assistance. This was followed by a medial closing wedge DFO with MPFL reconstruction, aiming to align the mechanical axis to pass through 70% of the tibial plateau, using a locking TriS plate (Olympus Terumo Biomaterials, Tokyo, Japan). Six weeks later, the same procedure was done for the right side (Figure 3). After six weeks of non-weight bearing on each operated side, he was allowed weight-bearing with the knee immobilized in extension with a brace for three weeks. Fifteen weeks after the second procedure, he was discharged home with full independence and returned to school life. Currently, he is independent in life including bicycle driving, without obvious ROM limitation on either side.

Physical function

Physical function parameters were assessed at each time point according to activity levels (Table 1).

**Table 1:** Change in physical function X, the day of the left-side operation; Y, the day of the right-side operation

	Pre-operative	X + 5 wk	Y + 2 wk (X + 8 wk)	Y + 4 wk	Y + 6 wk	Y + 12 wk	Y + 15 wk
ROM (deg; right/left)							
Knee flexion	90/95	--/60	50/70	60/75	75/70		110/85
Knee extension	-20/-10	--/-5	-15/-5	-15/-5	-15/-5		-15/-5
Gait ability							
Aids	Crutches	-	-	Crutches	Crutches	None	None
Comfortable speed (m/s)	0.68	-	-	0.32	0.60	0.70	1.03
Maximal speed (m/s)	-	-	-	-	-	-	1.25

The preoperative status included limited ROM on both sides and decreased walking speed with a requirement of crutches. Postoperatively, gradual improvements were observed on both sides; specifically, the right side showed relatively rapid recovery in flexion ROM compared with the left side despite a shorter postoperative duration, whereas the improvement in extension ROM was remarkable on the left side. Gait function improved throughout the inpatient rehabilitation period.

**Figure 4:**
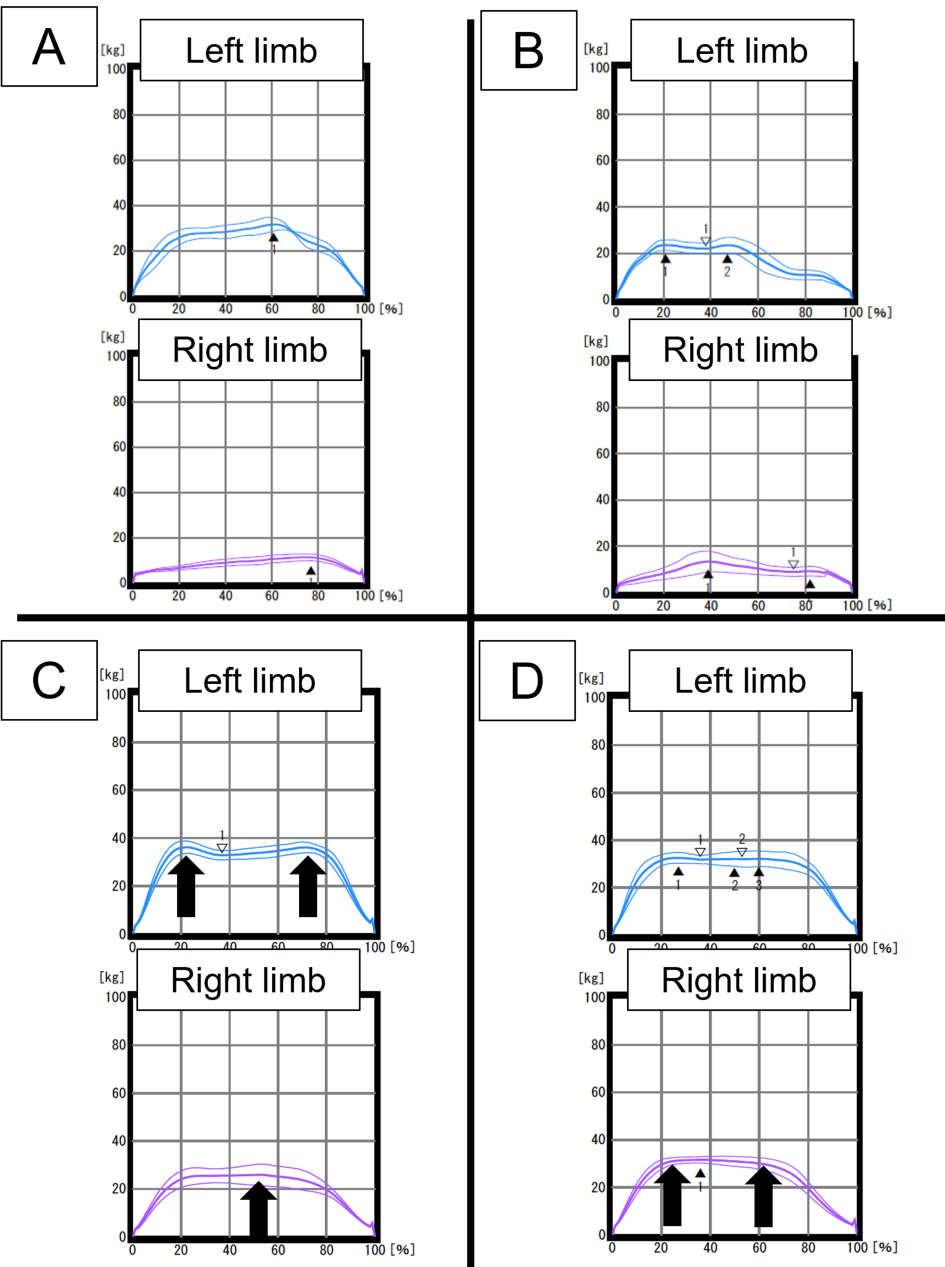
Load-time relationship during stance phase obtained using a shoe-type foot pressure measurement device The Y-axis represents the absolute load (kg) on each limb. The X-axis represents time normalized with the stance phase. Data were collected at seven, nine, 13, and 15 weeks after the second surgery for A, B, C, and D respectively. Gradual improvement in gait asymmetry (increase in right limb load) and gait normality (appearance of a pattern similar to the bimodal one) was observed, as indicated by arrows.

In addition, a detailed gait analysis was performed using a shoe-type foot pressure measurement device (MP-1000, Anima, Tokyo, Japan) seven weeks after the second operation to enhance the recovery of gait ability (Table 2, Figure 4). This device is equipped with two pressure sensors located in the forefoot and heel, capable of measuring plantar pressure at a sampling rate of 100 Hz. In this case, the following three parameters were used as indicators of gait symmetry and gait normality: average load during the stance phase (kg), maximum load (kg), and maximum load at the first peak (kg). The data were obtained from three continuous gait cycles during comfortable walking and expressed as the mean value.

**Table 2:** Gait analysis using a shoe-type foot pressure measurement device

	Y + 7 wk	Y + 9 wk	Y +13 wk	Y + 15 wk
Aids	Crutches	Crutches	None	None
Average load during the stance phase (kg; right/ left)	8.47/23.15	9.98/16.33	19.96/28.66	23.25/26.07
Maximum load (kg; right/ left)	13.34/34.32	19.30/27.49	33.23/39.93	34.14/38.21
Maximum load at the first peak (kg; right/ left)	-	13.50/23.48	25.91/36.21	31.63/32.47

Detailed gait analyses indicated an improvement in gait asymmetry (increase in right limb load) and gait normality (appearance of a pattern similar to the bimodal one).

Rehabilitation program

**Figure 5:**
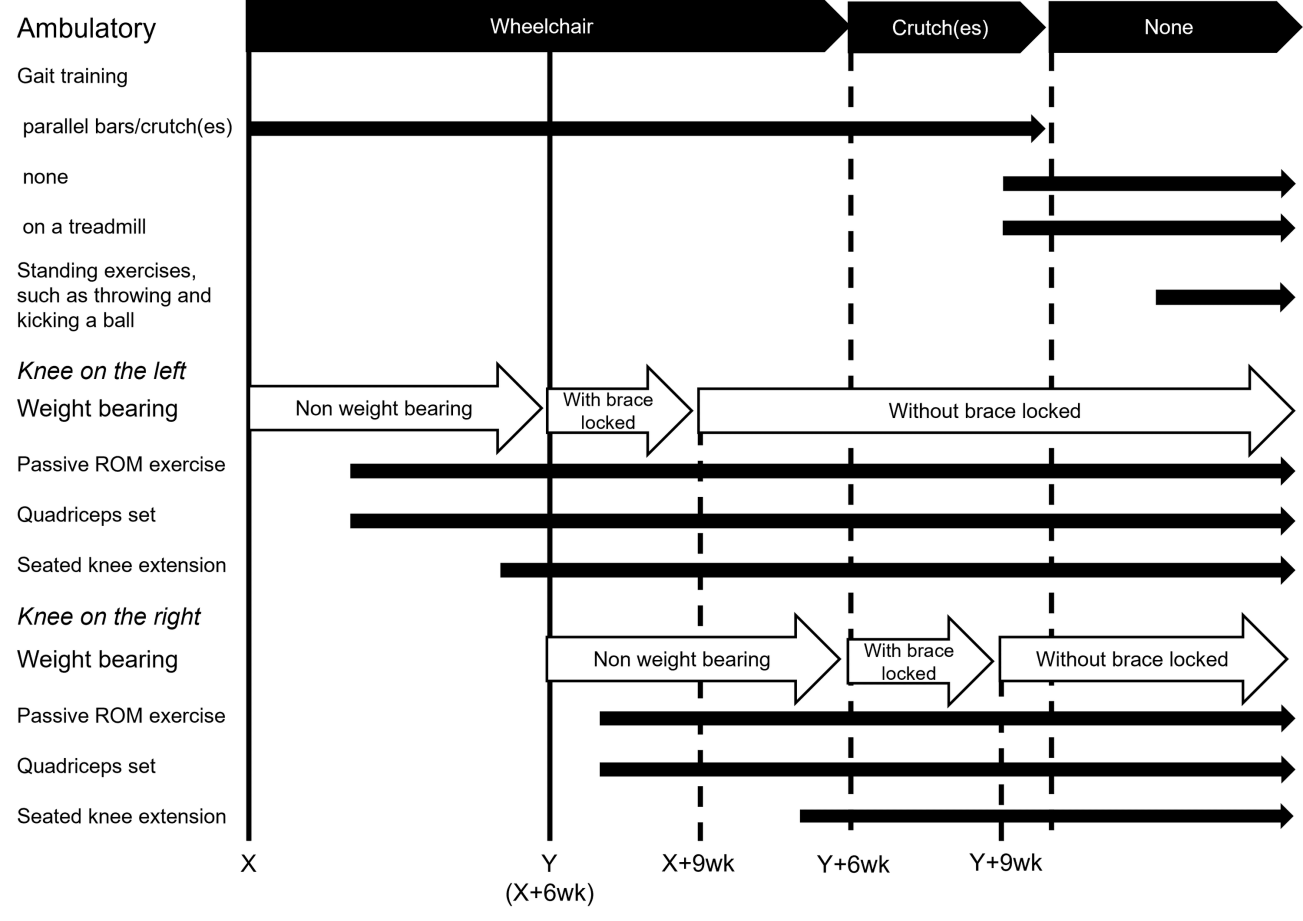
Rehabilitation protocol ROM, range of motion

The rehabilitation programs are shown in Figure 5. Comprehensive strength training, such as the upper limbs, core, and hip muscles was encouraged throughout the periods. In the left limb, passive ROM exercise and quadriceps set were started at two weeks postoperatively. The exercise intensity gradually increased, including seated knee extension, which was started at five weeks postoperatively. Postoperative rehabilitation of the right limb was performed in the same way as for the left limb, except that passive ROM exercise was started one week after the operation. The patient was unable to walk independently for 12 weeks due to non-weight bearing in either lower limb; gait training was only performed under supervision. Six weeks after the second operation, when weight-bearing with a locked brace was permitted, the patient was allowed to walk unsupervised with crutches. A further four weeks later, the patient was also allowed to walk independently without aid. At this time, however, a detailed gait analysis revealed two distinct abnormalities: left-sided dominant asymmetry and a flattened, non-bimodal pattern on both sides. To address these issues, several additional interventions were implemented, including real-time feedback of absolute load during gait using the aforementioned device, step exercises targeting specific phases of the gait cycle, and treadmill walking. Subsequently, standing exercises such as ball-throwing and kicking were introduced to further enhance lower limb loading.

## Discussion

We described a perioperative rehabilitation course and functional recovery following DFO in a case of patella dislocation accompanying femoral lengthening for ACH. Our results suggest that improvement in physical function, such as ROM and gait ability, could be achieved via DFO and postoperative rehabilitation, even in cases with poor physical function and disease-specific limb deformities. To the best of our knowledge, this is the first report to describe the post-DFO functional recovery in an ACH patient with patella dislocation following femoral lengthening.

We observed a post-DFO improvement in physical function in a complex case characterized by disease-specific and treatment-related factors. The specific characteristics of this case were ACH as a congenital disorder, a history of limb lengthening in the tibia and femur, and the resulting preoperative poor physical function. Although the efficacy of DFO combined with MPFL reconstruction for valgus deformity has been well recognized, little is known in such complex cases. In this case, improvement in ROM and gait ability was achieved compared to preoperative levels, except for flexion ROM on the left side. Disease-specific joint laxity can be considered as one of the potential reasons for the relatively early recovery in ROM. Those with ACH commonly exhibit joint and soft tissue laxity [6,15]. This characteristic may have contributed to the early recovery in ROM accompanying correcting valgus knee deformity. On the other hand, one contributing factor to the residual ROM limitation may be soft tissue contracture. In this case, preoperative ROM limitation was assumed to involve not only valgus knee deformity but also soft tissue conditions related to femoral lengthening and quadriceps tightness, which was associated with the permanent dislocation of the patella. Joint contracture is a well-observed complication during and even several months after limb lengthening [6,16]. Moreover, it takes a long time, more than several months, to increase muscle extensibility [17]. Therefore, these mechanical changes were not observed in acute inpatient rehabilitation. Fortunately, no obvious ROM limitations were observed at the one-year follow-up.

Gait ability such as lateral symmetry and load pattern was improved via detailed gait analyses and extended programs, both with the use of a shoe-type foot pressure measurement device. We used a shoe-type foot pressure measurement device for the assessment and intervention of gait abnormalities. These assessments revealed qualitative abnormalities presented visually in the load-time relationship, as well as quantitative abnormalities, such as lateral asymmetry. These parameters are crucial for gait energy efficiency [18]. Moreover, we tried to improve these parameters through extended rehabilitation programs, including the use of the device. As a result, an increase in the absolute load on the right side and an improvement in the load pattern on both sides were achieved. These results suggest the effectiveness of tailor-made rehabilitation approaches in such complex cases. While short-term improvements were observed, further studies are needed to evaluate their impact on long-term outcomes.

## Conclusions

This report highlights that perioperative rehabilitation combined with DFO is effective in improving physical function, including ROM and gait ability, in ACH patients with severe functional impairments and characteristic limb deformities. These results provide valuable insights into managing complex cases and underscore the potential of DFO and targeted rehabilitation to enhance patient outcomes. Further studies are required on the rehabilitation strategies to maximize functional recovery in such cases.
